# Telemetric electroencephalography recording in anesthetized mice—A novel system using minimally-invasive needle electrodes with a wireless OpenBCI™ Cyton Biosensing Board

**DOI:** 10.1016/j.mex.2023.102187

**Published:** 2023-04-15

**Authors:** Mohammad T. Mansouri, Meah T. Ahmed, Tuan Z. Cassim, Matthias Kreuzer, Morgan C. Graves, Thomas Fenzl, Paul S. García

**Affiliations:** a Department of Anesthesiology, Vagelos College of Physicians and Surgeons, Columbia University, New York, NY 10032, USA; b Department of Anaesthesiology & Intensive Care, School of Medicine, Technical University of Munich, Munich, Germany

**Keywords:** Electroencephalography, Telemetry, Subdermal needle electrode, Isoflurane, Anesthesia, Burst suppression, Telemetric Electroencephalography Recording in Anesthetized Mice Using Minimally-invasive Needle Electrodes with A Wireless OpenBCI TM Cyton Biosensing Board

## Abstract

Telemetric electroencephalography (EEG) recording, using subdermal needle electrodes, is a minimally-invasive method to investigate mammalian neurophysiology during anesthesia. These inexpensive systems may streamline experiments examining global brain phenomena during surgical anesthesia or disease. We utilized the OpenBCI™ Cyton board with subdermal needle electrodes to extract EEG features in six C57BL/6J mice undergoing isoflurane anesthesia. Burst suppression ratio (BSR) and spectral features were compared for a verification of our method. Following an increase from 1.5% to 2.0% isoflurane, the BSR increased (Wilcoxon-signed-rank statistic; p = 0.0313). Furthermore, although the absolute EEG spectral power decreased, the relative spectral power remained comparable (Wilcoxon-Mann-Whitney U-Statistic; 95% CI exclusive AUC=0.5; p < 0.05). Compared to tethered systems, this method confers several improvements for anesthesia specific protocols: 1-Avoiding electrode implant surgical procedures, 2-Anatomical non-specificity for needle electrode placement to monitor global cortical activity representative of anesthetic state, 3-Facility to repeat recordings in the same animal, 4-User-friendly for non-experts, 5-Rapid set-up time, and 6-Lower costs.

• Minimally-invasive telemetric EEG recording systems ergonomically improve tethered systems for anesthesia protocols.
• Using this method, we verified that higher isoflurane concentrations resulted in an increased EEG burst suppression ratio and decreased EEG absolute spectral power, with no change in frequency distribution.

## Graphical abstract

**Figure:**
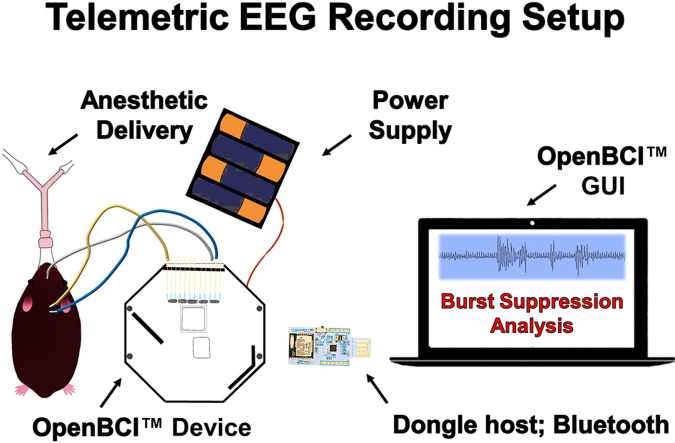

Specifications table

**Table:** 

Subject area:	
More specific subject area:	Neuroscientific laboratory and anesthesia research
Name of your method:	Telemetric Electroencephalography Recording in Anesthetized Mice Using Minimally-invasive Needle Electrodes with A Wireless OpenBCI™ Cyton Biosensing Board
Name and reference of original method:	Mang, G. M., Franken, P. (2012). Sleep and EEG phenotyping in mice. Current Protocols in Mouse Biology, 2, 54–74. 10.1002/9780470942390.mo110126
Resource availability:	N/A

## Method details

Within the last few decades of using *in vivo* approaches in the laboratory, telemetry has become widely used to measure a variety of behavioral and physiological parameters in animals of various sizes. Prior to telemetry, tethered systems were the most convenient method for recording neurophysiologic data such as electroencephalographs (EEG) in pre-clinical models [1]. Typically, these systems required survival surgery for which electrodes are connected to a miniature socket anchored to the skull of the animal. The sockets were then connected to data acquisition devices via cables. Several authors, from various neuroscientific foci, have described tethered recording systems as a semi-restraint model with negative implications for animal welfare [[1], [2], [3], [4], [5], [6]]. The ergonomic disadvantages to such systems are their long setup times, which involve lengthy surgical procedures, and spatial organization of cables that risk entanglement. Furthermore, with regards to neuroscientific studies that require natural movement (e.g., sleep), the use of length-limited or heavy cables can reduce the animal's mobility if not specifically counterbalanced with elaborate commutators. Another disadvantage of tethered systems, especially for surgical anesthesia neuroscience investigations, is the risk of surgical complications such as bleeding or infection at the electrode implantation site. Hemodynamic instability and necrosis caused by surgical infections can affect the health of housed animals, or the collected experimental data. And when severe, these complications may result in euthanization of the animal for compassionate reasons and premature removal from the study. Sometimes, elaborate experimental designs require accounting for these potential subtractions from animal cohorts and working with these larger sample sizes can be a financial disadvantage. Telemetric wireless data acquisition systems override some of these challenges and should be utilized in neuroscientific investigations of anesthesia [or other neuroscience specific protocols investigating EEG].

Several “turn-key” telemetry EEG methods are available for rodent monitoring (i.e., Epoch, multichannel systems, DSI, etc.) [[7], [8], [9]]. Such systems allow for monitoring and recording of physiologic data at a distance without hindering the animal's natural mobility. While telemetry is already a well-established improvement to traditional restraining procedures for EEG monitoring, developing even simpler (e.g., non-implantable) methods can confer advantages for neurophysiologic investigations of anesthesia where whole-brain phenomena are present. Streamlining EEG monitoring may also aid several other major neuroscientific areas of research such as investigations surrounding sleep, cerebrovascular disorders, encephalitis, traumatic brain injury, brain tumors, epilepsy, Alzheimer's disease, etc.

EEG represents the electrical structure of the synaptic activity of the cerebral cortex, as reflected by its apically oriented cortical neuronal populations (i.e., mostly layer 5 pyramidal neurons) [10,11]. Changes in brain activity, in some cases, can be represented as a significant shift in EEG spectral information. For example, as reported by Harris et al., selective attention in rodents can be characterized as a modulation of low-frequency activity fluctuations and spiking correlations throughout the cortex [11]. The electrodes required to measure global cortical changes do not always require anatomical specificity and precision; in other words, because EEG monitors global brain activity, the location of the electrodes need not be absolutely strict to collect this operational data contained in biologically relevant frequency bands. Needle electrodes placed generally anywhere between the skull and the scalp, sub-dermally, adjacent to any approximate cortical landmark with expected electrophysiological changes (i.e., between the nasal and frontal bones) will permit quality EEG recordings for the same animal even when removed and re-applied with care; in fact, in clinical models, it is often preferred for direct cortical recording [12]. The safety and efficacy of such sub-scalp and sub-galeal electrodes for electrophysiological signal recordings is well-established by various authors [[13], [14], [15]]. Despite the skull attenuating high-frequency signals, it has been reported that these electrodes can record high-frequency signals up to 30-100 Hz without frequency distortion [14,16]. The use of needle electrodes as a minimally-invasive method to monitor EEG in rodents is a reliable translational tool that has a primary focus on anesthesia research. It eliminates the need to perform elaborate survival surgeries on the skull, thus allowing for anesthesiology investigators lacking specialized expertise to monitor EEG during quiescent states

In the realm of anesthesia, EEG patterns can be used to assess brain health and the overall pharmacodynamic effects of anesthetic drugs on brain electrophysiology [17]. Selected EEG parameters are of critical interest in the implication of anesthesia's effect on global brain circuit oscillatory patterns. For instance, burst suppression (BS) is one such EEG pattern characterized by alternating periods of high amplitude activity (bursts) and near isoelectric signals (suppressions). Namely, BS is a discontinuous EEG pattern characterized by patterns of near electrical silence (<10 μV amplitude for periods of >1 s) intermixed with episodic bursts of neural activity defined as brief (0.5 - 5 s) epochs of moderate frequency EEG activity (2 - 20 Hz) of sufficient amplitude (>25 μV peak to peak) [18]. This global neurophysiological phenomenon is not present during natural sleep in healthy brains. It can, however, arise from administration of high-dose hypnotic agents used for anesthesia care and in several different pathological conditions including hypoxemia, coma, and toxic doses of ethanol, baclofen, carbamazepine, bupropion, etc. [19]. A powerful EEG feature for decision-making in the clinical setting, BS is a treatment for refractory status epilepticus [20]. Although a growing body of evidence demonstrates that volatile and intravenous anesthetics may have distinct electrophysiological patterns during BS [19], no standard methodology of evaluating BS exists for rodent models undergoing specific anesthesia regimens. Until now, evaluation of BS in pre-clinical models has not been demonstrated, particularly with a more simplified and minimally-invasive EEG monitoring method.

In this neuroanesthesia study, we described a telemetrically configured needle electrode system to record EEG and characterize the BS patterns in mice induced by a common general anesthetic (isoflurane). We also demonstrated that by using our telemetric needle electrode system, offline spectral analysis may be performed on the collected EEG data for assessment of changing EEG frequency band activities due to the effects of isoflurane on the global brain electrophysiology. In doing so, we verified that EEG-based neuromonitoring for surgical anesthesia protocols in rodents can be made more ergonomic and less-invasive as compared to tethered EEG recording systems. Our system has a significantly lower cost, facilitates multiple recordings in the same animal during complex surgical anesthesia protocols (i.e., inserting and re-inserting needle electrodes as needed), allows for natural animal movement during anesthesia emergence, and allows for use by researchers with minimal specialized neurophysiologic expertise.

### Experimental set-up & anesthesia protocol: isoflurane-induced burst suppression recording

**Fig. 1:**
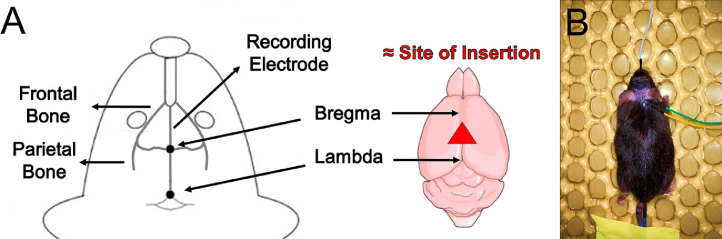
Anatomical reference for EEG recording electrode insertion and the animal's placement. After induction and LORR of the animal, three electrodes were inserted following local anesthetic administration; (A) The insertion of the recording electrode was anteromedial from bregma along the frontal bone. The recording electrode captured cortical activity from the frontal lobe. The approximate location of the recording electrode is depicted in a red transparent wedge. (B) Photo representation of example experimental setup. The white insulated wire is the recording electrode. The two reference EMG electrodes [yellow and green] were inserted over the muscles of the neck. The blue wire is the rectal temperature monitor.

A total of 6 age-matched adult male (3-4 months old, 26-30 g) C57BL/6J mice were used. The mice were group housed, kept on a 12-hour alternating zeitgeber light/dark cycle, and allowed access to food and water *ad libitum*. Briefly following 3 days of handling and habituation, the animals were transported to the laboratory for EEG recording during an anesthesia induction and maintenance protocol. On the day of each experiment, the mouse was anesthetized with inhaled isoflurane (4% isoflurane in 1.5 L/min of pure oxygen) using a vaporizer in a pre-filled isoflurane (Covetrus, India) anesthesia induction chamber. To assess for loss of righting reflex (LORR) following induction, the animal was checked for responsiveness to toe-pinch. The animal was subsequently transferred to a heated pad in a prone position, after which the animal was immediately placed in a nose cone for continued isoflurane delivery and anesthesia maintenance. The animal was kept normothermic (between 36.5 and 38 °C) by means of a warm-water circuit (HTP-1500, Adroit Medical System). Before insertion of the needle electrodes, eye lubricant ointment was applied to prevent eye dryness. Furthermore, to facilitate electrode placement under the animals’ skin with minimal discomfort, a local anesthetic of 0.05 mL bupivacaine (0.25% concentration, Hospira, IL) was applied prior to insertion. Then, using forceps for skin countertraction, a Rhythmlink™ subdermal needle electrode (Rhythmlink, SC, USA) was inserted into the subcutaneous space over the scalp of the frontal cortex in between the animal's eyes, and two reference electromyogram (EMG) electrodes were inserted into the right neck muscle approximately adjacent to each other. These subdermal needle electrodes consisted of sharp 5 – 7 mm tip lengths and 0.5 mm needle diameters (Rhythmlink, SC, USA). This differential electrode configuration helped avoid contamination of the EEG signal from physiological artifacts (i.e., cardiac activity, myogenic noises) as much as possible. An approximate anatomical reference for insertion of the recording electrode, as well as a photographed example of the situated animal, can be seen in Fig. 1A and 1B, respectively. The recording electrode was inserted over the frontal cortex, with the intention of capturing general cortical activity. Moreover, the placement of the reference EMG electrodes is viable for any region around or within the left or right neck muscles, where muscle or cardiac-induced physiological artifacts are most prevalent.

The dose of anesthesia maintenance after induction and transfer of the animal to the nose cone was initially set to 1.5% isoflurane in oxygen (fresh gas flow rate of 1 L/min). As aforementioned, insertion of the EEG needle electrodes was performed after transitioning to the nose cone for anesthesia maintenance. Typically, the manipulation of animals and wires was complete within 10 min of anesthesia induction, after which EEG was recorded for approximately 15 min at 1.5%. Thereafter, the isoflurane maintenance dose was increased from 1.5% to 2.0%, and the EEG data collection continued for another 10 min period. For each animal, we acquired 25 min of EEG recording, capturing two changing concentrations of isoflurane. Each animal was used only once for the recording. Physiological parameters such as body temperatures (°C) and respiratory rates (breaths/min) were monitored at 5-min intervals throughout the experiment.

### EEG recording with the OpenBCI™ device and Rhythmlink™ needle electrodes: specifications

The telemetric EEG signals analyzed in our method were acquired wirelessly using the OpenBCI™ 8-channel Cyton Biosensing Board (OpenBCI, NY, USA). The subdermal needle electrodes (Rhythmlink, SC, USA) were connected as analog inputs to the board. For EEG recording in mice, we found that subdermal needle electrodes are advantageous for anesthesia specific protocols. They are unchallenging to place and suitable for short-term EEG recording (<30–40 min). Furthermore, in comparison to surgically situated EEG implants, we verified that investigators may repeat recordings multiple times in the same animal by inserting, removing, and re-inserting the needle electrodes as needed, without hampering the quality of the signal; When neuromonitoring during a complex surgical anesthesia induction and maintenance protocol, the convenience of removing and re-inserting the electrodes as needed for protocol specific tasks (i.e., mouse positioning changes or site-specific injections) makes the use of subdermal needle electrodes superior. The use of needle electrodes also allows for the investigator to modify/customize the system as desired (i.e., clipping the needle electrodes at an angle for smaller animals). However, investigators may choose to utilize electrodes of different lengths that are available in the market, such as 7 mm tip length subdermal needles for mice or 13 mm subdermal needles for rats (Rhythmlink, SC, USA).

**Fig. 2:**
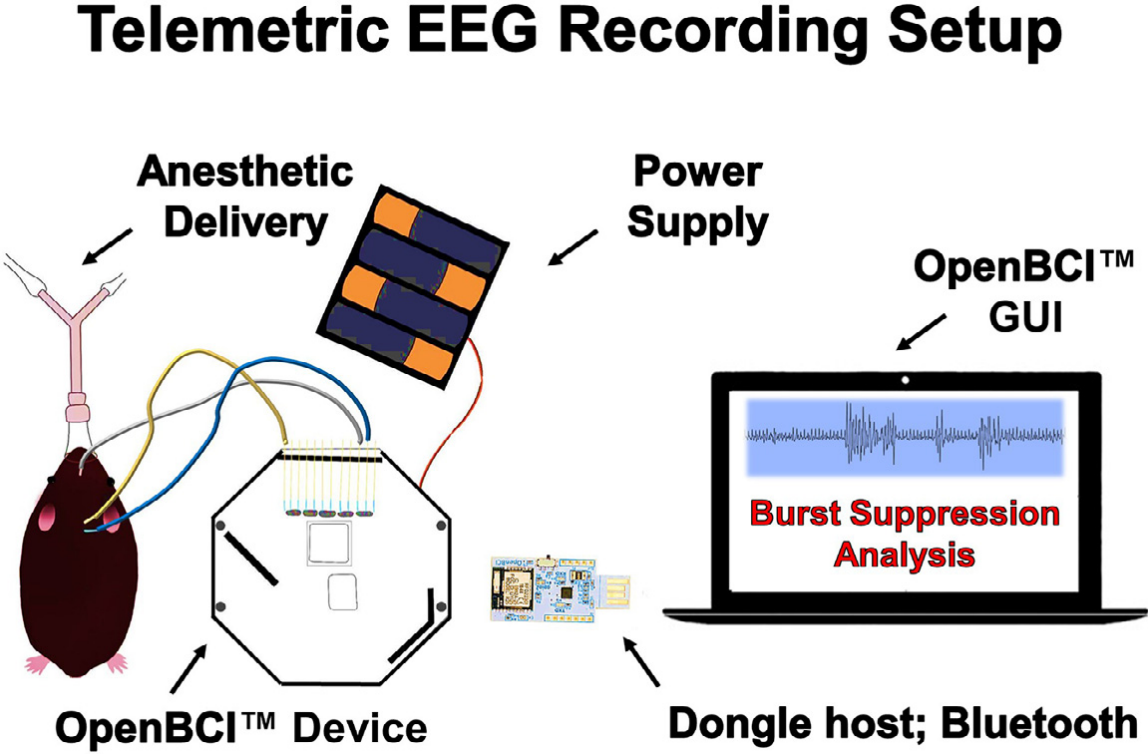
Telemetric EEG recording using needle electrodes and OpenBCI™. Mice under isoflurane anesthesia were situated for EEG recording via subdermal needle electrodes integrated with the OpenBCI™ 8-channel Cyton Biosensing Board. The device contains various hardware features such as a Texas Instruments© ADS1299 Analog-to-Digital Converter (Texas Instruments, TX, USA). The processed raw EEG data is wirelessly transmitted through RFduino™ Low Power Bluetooth™ radio transmission signals and received at the USB dongle host on a computer node. The EEG signals are then stored and visualized at the computer node via *the data acquisition, processing, and design tool OpenBCI™ graphical user interface*.

The raw EEG data obtained at the Cyton Biosensing Board node is converted from analog-to-digital format at a sampling frequency of 250 Hz. A benefit of choosing such an open-source design as the operating system node is such that an investigator may tinker with the sampling frequency as desired (i.e., to optimize EEG resolution). Nevertheless, the recorded raw EEG data is then transferred to the computer's USB slot through Bluetooth low energy radio transmission and saved as CSV, TXT, or other formattable files for analysis. The Cyton Biosensing board (the device) performs this transmission by communicating with the OpenBCI™ USB radio receiver dongle (the host). For visual interpretation and control of signal acquisition, live raw EEG signals can be seen on the computer [where data is received] through the data acquisition, processing, and design tool OpenBCI™ graphical user interface (GUI). This allows for the investigator to keep the pre-amplifier close to the animal, but the recording node (i.e., laptop computer) far away from the animal; this holds two major advantages which are 1) minimizing the amount of electrical noise close to the needle electrode sensors (i.e., noise from large computers) and 2) allowing for remote monitoring/analysis. This setup is shown in Fig. 2.

## Data analysis and signal processing

We used MATLAB R2019a (The Mathworks, Natick, MA, USA) for our analyses. A custom MATLAB script was used to visualize the EEG data from all 6 mice. The EEG data subjected to analyses were 1) full-length recordings of the net 25 min period, 2) 20 s EEG episodes with minimal line noise and artifact representative of anesthetic equilibration from both the 1.5% and 2.0% isoflurane concentrations, and 3) 3 min recordings from both concentrations that best represented the severity of discontinuous EEG (i.e., BS).

Before episode extraction, all of the EEG data were band-pass filtered with a Butterworth filter, to the 0.1 to 30 Hz range, using a MATLAB-based zero-phase shift routine (filtfilt). Furthermore, a 60Hz notch filter was utilized to remove the noise of electrical outlets. Spectral analysis was performed, on the full-length recordings, as well as on the 20 s episodes. Spectral powers were calculated using the pwelch function of default settings, and a non-uniform fast Fourier transform (NFFT) of 512, leading to a frequency resolution of 0.5 Hz. The absolute and relative (normalized) power spectral density plots (PSD) were calculated from the 20 s episodes; the relative PSD is defined as the ratio of the PSD to the frequency band to be analyzed and the total frequency band. The density spectral arrays (DSA) [or also known as spectrograms] from the full-length recordings were calculated for EEG windows of 10 s lengths with a 1 s shift.

The 3 min time-series EEG episodes were subjected to single-blinded burst suppression ratio (BSR) analysis. In this analysis, in order to identify and isolate BS, a visual sweep of each [untagged] 3 min EEG episode was speculated for peak-to-peak amplitudes of burst epochs vs. suppression epochs by the blinded examiner. As a standard to determine an *amplitude threshold* for the suppression events that preceded the bursts of BS (i.e., a threshold to define the isoelectricity of BS), a 3/10th factor was applied; for example, in animals with noisier EEG (i.e., line noise or respiratory/cardiac artifacts), if bursts were seen to reach amplitudes >50 µV, the standard threshold for suppression was considered as any epoch of signal with peak-to-peak amplitudes <15 µV lasting for at least 100 ms. On the contrary, in animals with EEG that were more quiescent or more affected by the changing anesthesia dose (i.e., signal suppression), if bursts were seen to reach lower amplitudes (e.g., approximately >30-to-35 µV), the standard threshold for suppression was considered as any epoch of signal with peak-peak amplitudes <10 µV lasting at least 100 ms. For each episode, the collective time sum of suppression epochs was divided by the total EEG epoch duration, thus resulting in a BSR value. Unfortunately, the EEG signals could not all employ a singular standard to decide an *amplitude threshold* that identifies suppression epochs, because electromagnetic or physiological noise/artifacts impacted all of the EEG recordings’ signal-to-noise ratio [and overall signal quality] heterogeneously. Nonetheless, an automated amplitude-based BS detection cannot be utilized using our method: this procedure of BSR identification is thus a subsequent limitation for our proposed method.

Collection of the physiological parameters was also subjected to basic graphical analysis. The body temperatures (°C) and the respiratory rates (breaths/min) for all 6 mice were visualized as box plots.

### Statistical analysis

The PSD calculations across the 20 s EEG episodes were subjected to an area under the [receiver operating] curve (AUC) calculation from the Wilcoxon-Mann-Whitney U-Statistic. We conducted the AUC calculation with 10-k fold bootstrapped 95%-confidence intervals using the MATLAB-based MES toolbox. A 95% confidence interval exclusive AUC=0.5 indicated significance at p < 0.05 [21]. Distribution of AUC values for each frequency mean rank was visualized in a plot. To consider increased chances of false positives due to multiple comparisons, we only discussed results as significant if the significant differences occurred in neighboring frequencies. This approach was established before, as previously reported by other authors [22,23]. The BSR analysis across the 3 min EEG episodes was subjected to a Wilcoxon signed-rank test. We performed the calculation with a 95% confidence interval, thus indicating significance at p < 0.05. Lastly, no statistical analysis was performed on the physiological parameters: significance was concluded only based on visual differences of their parameters, so we only used descriptive statistics to present and discuss the results of the monitored physiological parameters.

## Method results

**Fig. 3:**
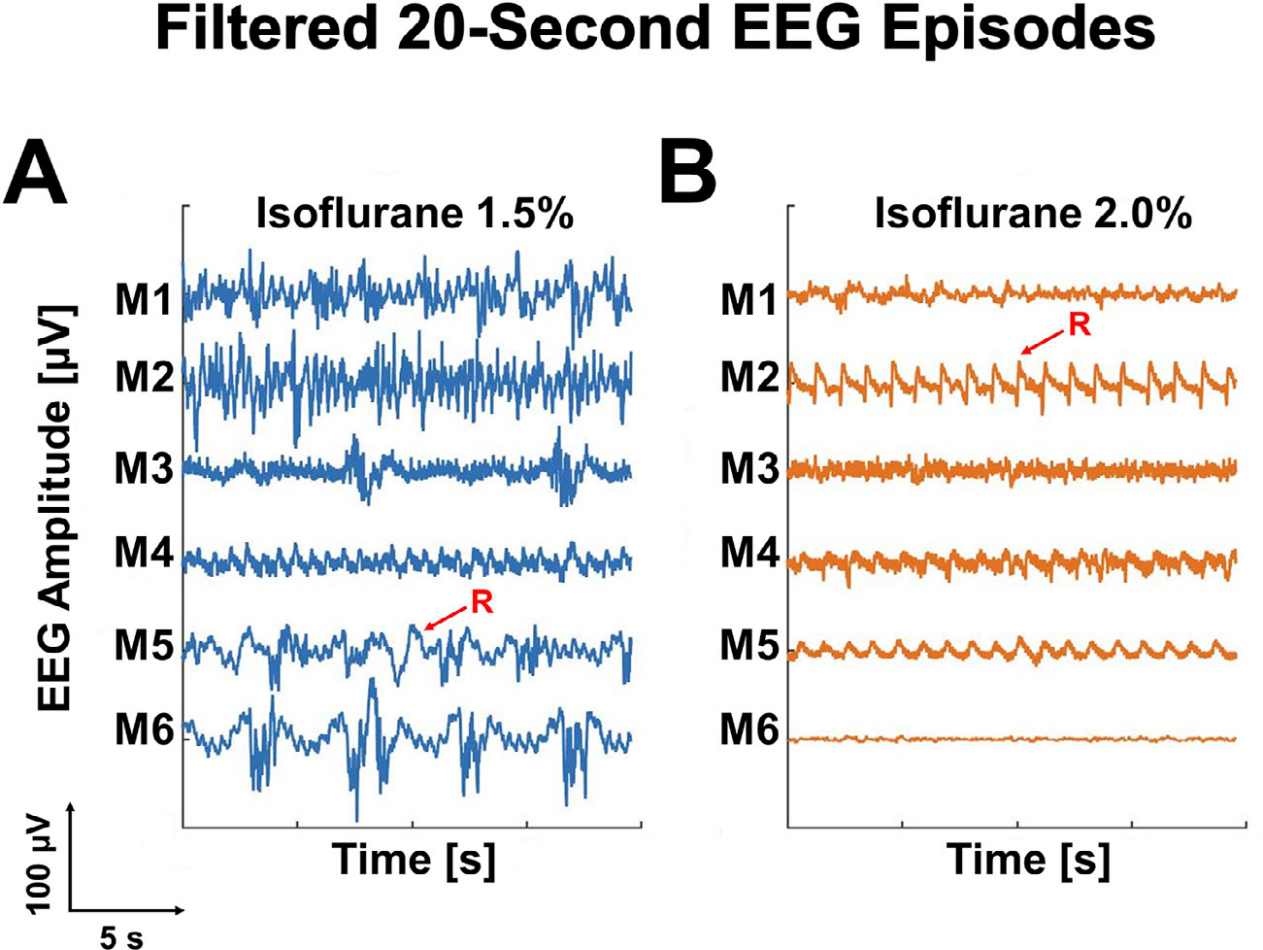
Filtered 20 s EEG episodes between two concentrations of isoflurane. Time-series EEG traces from all mice under 2 different isoflurane concentrations; administering maintenance doses of 1.5% isoflurane (A, left panel, blue) exhibits higher energy in low and moderate frequency ranges as compared to 2% isoflurane (B, right panel, orange). Respiratory artifacts are marked with a bolded red ‘R’.

Recorded and processed EEG signals from all six mice were interpretable as functional global electrical activity of the animal's cerebral cortex. Shown in Fig. 3a and 3B are extracted 20 s EEG episodes for both isoflurane concentrations. Despite filtering, some physiological artifacts such as movement, cardiac, or respiratory artifact can still be observed in the traces; respiratory artifacts were more easily seen in the suppressed EEG traces during 2.0% isoflurane maintenance (Fig. 3b). Nevertheless, high amplitude and high frequency signals were suppressed after increasing the isoflurane concentration from 1.5% to 2.0%. When using this method, witnessing the transition from continuous EEG traces at a broad frequency range to a discontinuous near isoelectric signal with increasing anesthetic concentration is the most reliable way to determine your signals are generated by brain activity.

**Fig. 4:**
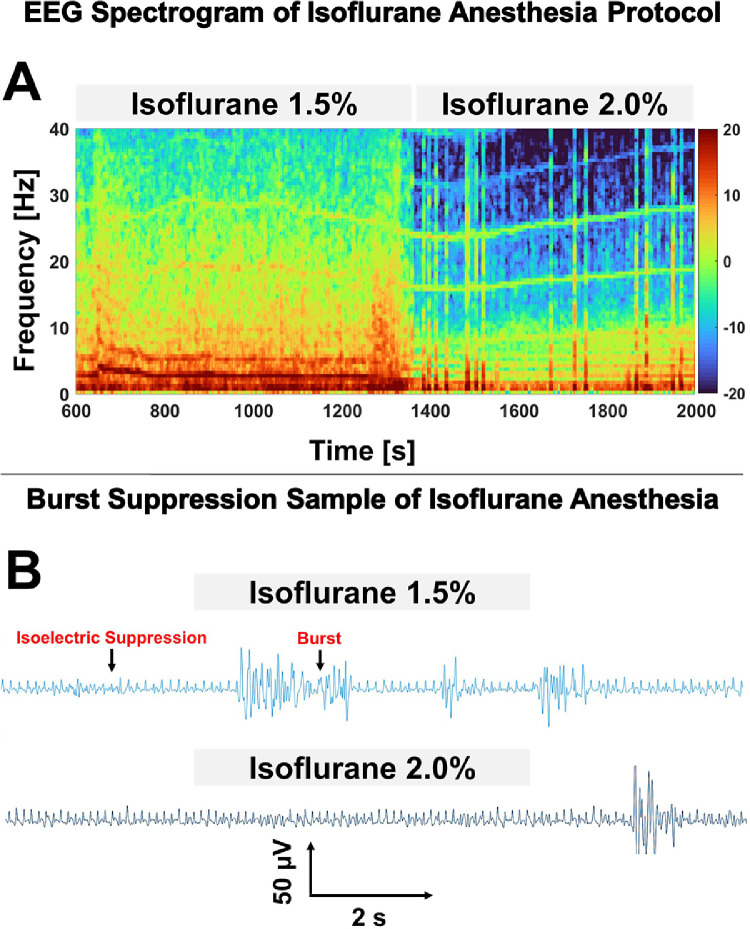
Spectral and visual burst suppression characteristics of EEG recorded with needle electrodes configured using OpenBCI™ telemetric data acquisition. (A) EEG Spectrogram derived from telemetric EEG recording with needle electrodes; the spectrogram represents the changes in frequency distribution for the 25 min continuous recording of EEG for one animal subjected to two different isoflurane concentrations. With an increase in drug concentration, there was a general decrease in EEG power over the entire frequency range. Burst suppression was also observed during delivery of both isoflurane concentrations but was more evident with 2.0% isoflurane. Burst suppression in the spectrogram is shown as periods of blue (isoelectric suppression activity) interspersed with periods of red-yellow (delta and theta oscillations). This verified that this method can monitor viable EEG traces and its changes due to the effects of anesthetic hypnosis. From the spectrograms, narrow-band artifacts can also be observed due to electrical resonance throughout the experiment. (B) Examples of burst suppression between the 1.5% and 2.0% isoflurane groups detected with telemetric EEG recording with needle electrodes; 12 s EEG traces depict representative examples of bursting epochs discontinuously separated by periods of suppression (near isoelectricity). Suppression epochs preceding the bursts of burst suppression during delivery of 2.0% isoflurane were generally longer than the suppression epochs during delivery of 1.5% isoflurane. Furthermore, respiratory artifacts are present and easier seen during periods of isoelectric suppression.

The 25 min total EEG can be represented as time-varying comparative distributions of the energy in different spectral bands. Subsequently, the generated DSA [or spectrogram] can clearly track spectral changes of the animal's EEG due to changing concentrations of 1.5% to 2.0% isoflurane. A sample DSA can be seen as shown in Fig. 4a. During delivery of 1.5% isoflurane, the EEG contained significant oscillations in the alpha (8-12 Hz) and delta (1-4 Hz) bands, while a relative paucity of oscillatory EEG power due to BS was observed in the presence of 2.0% isoflurane. This change in spectral bands affirmed that EEG was prevalent in our signal, despite artifact contaminations. As also seen in the DSA, several horizontally oriented artifacts are evident by their narrow frequency band. These represent electromagnetic resonance from the animal's pulse and/or nearby devices. Nevertheless, shown in Fig. 4b are also extracted 12 s samples of EEG traces with the presence of BS. It was clearly observed that, compared to the BS in EEG during delivery of 1.5% isoflurane, the suppression epochs of BS during delivery of 2.0% isoflurane were longer.

**Fig. 5:**
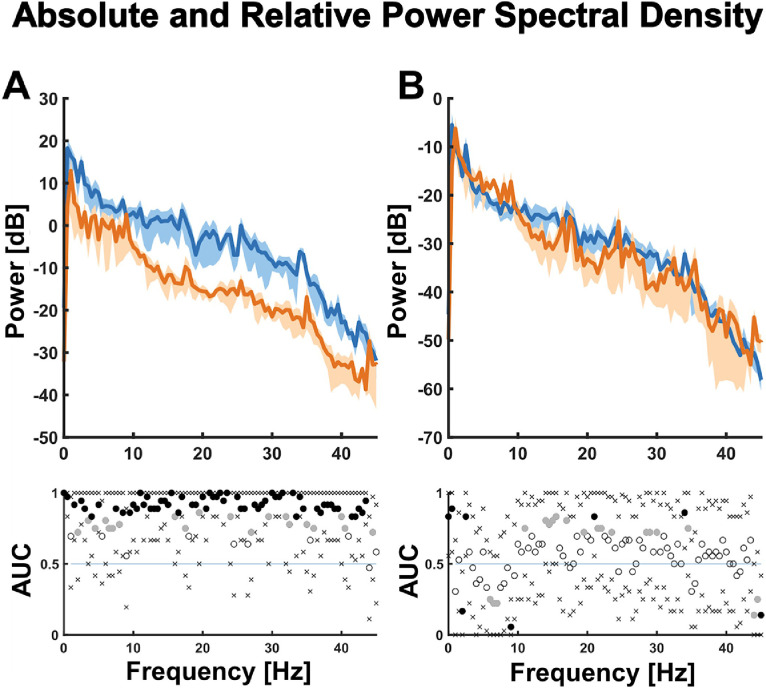
Absolute and relative power spectral densities from 20 s EEG episodes between two maintenance concentrations of isoflurane. (A) Absolute power spectral densities between two isoflurane anesthesia maintenance concentrations; at 1.5% isoflurane anesthesia maintenance (blue), the EEG had significantly higher power over the entire frequency range, compared to the EEG at 2.0% isoflurane anesthesia maintenance (orange). (B) Relative power spectral densities between two isoflurane anesthesia maintenance concentrations; the overall architecture of the EEG, as assessed by the relative power, was not significantly different between the 1.5% and 2.0% isoflurane groups.

Fig. 5 shows absolute (5A) and relative (5B) PSD plots for the two anesthesia maintenance concentrations (1.5% and 2.0% isoflurane). The overall (absolute) power of the EEG signal decreased after transitioning to 2.0% isoflurane. At the same time, the relative distribution of power among all EEG frequency bands (e.g., delta, theta, alpha, etc.) was not significantly different between the 1.5% isoflurane and 2.0% isoflurane groups.

**Fig. 6:**
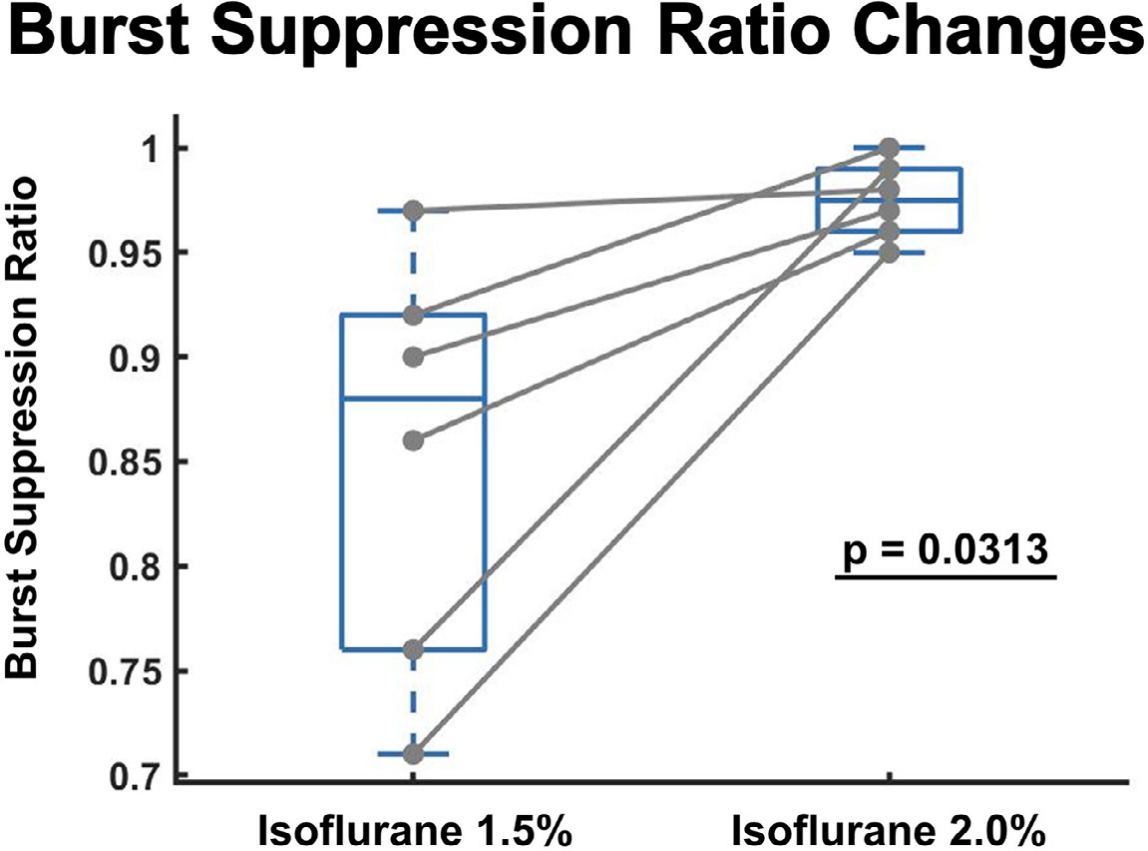
Comparing burst suppression ratio in mice receiving 1.5% isoflurane followed by 2.0% isoflurane for anesthesia maintenance. The burst suppression ratios were based on visual inspection of EEG data by raters blinded to experimental conditions. The burst suppression ratios increased in every animal after an increase in isoflurane dose (p<0.05).

Fig. 6 demonstrates the analysis of BSR. In every tested animal, receiving 2.0% isoflurane anesthesia for anesthetic maintenance resulted in a greater BSR as compared to 1.5% isoflurane. This indicates that there were longer periods of EEG discontinuity (durations of near electrical silence and isoelectric suppression) in the higher isoflurane concentration, with greater latencies between bursts. A "ceiling" effect in burst suppression ratios is also evident; as it is impossible to have a BSR > 1.0, low variance is observed in the 2.0% isoflurane group, and any animal with a high BSR during maintenance at 1.5% isoflurane will have a comparatively smaller increase in BSR during exposure to 2.0% isoflurane indeed.

**Fig. 7:**
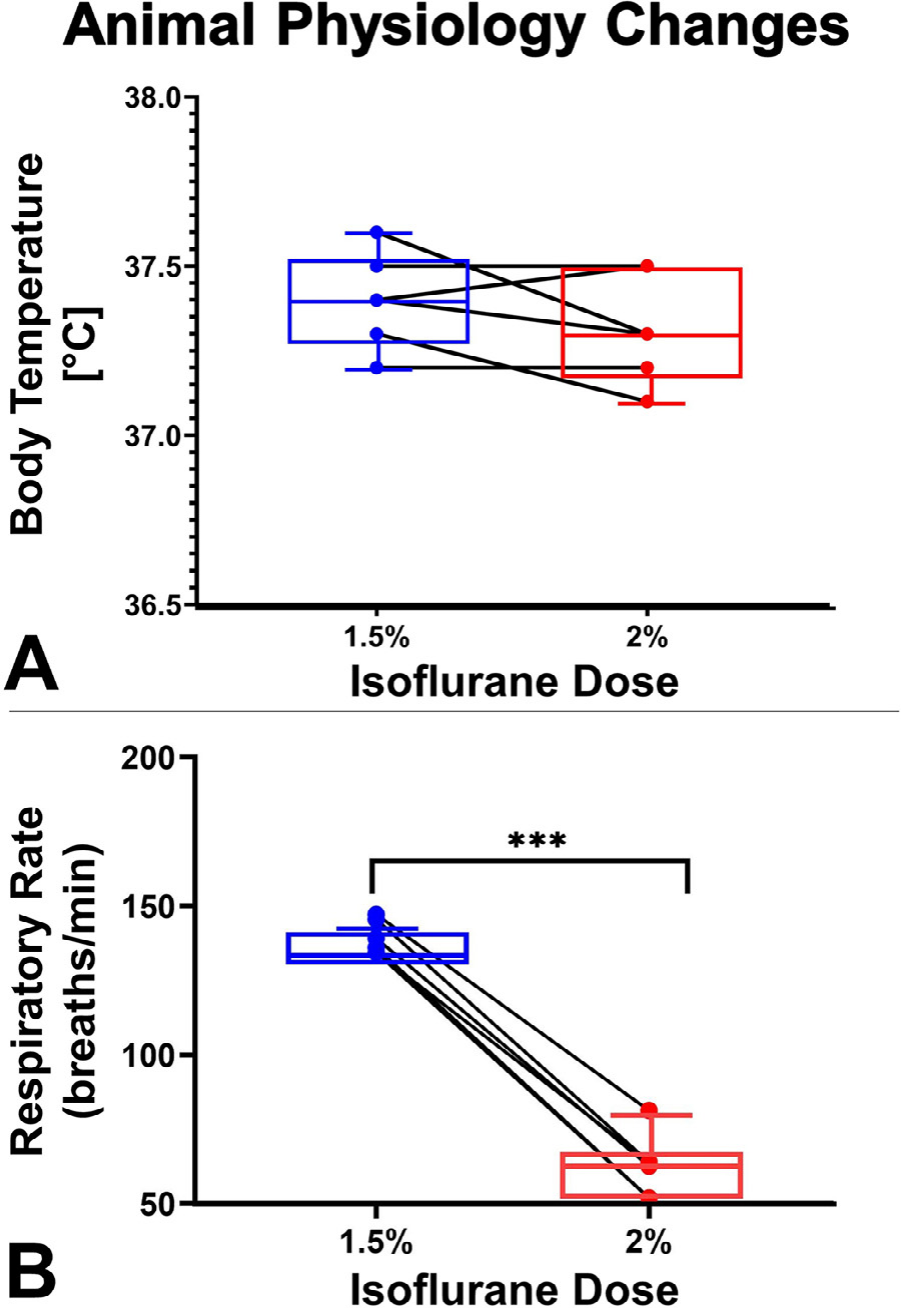
Effects of two isoflurane concentrations on physiologic markers. Body temperatures (°C) and respiratory rates (breaths/min) were collected at 5 min intervals throughout isoflurane delivery. (A) The body temperatures of all 6 mice were kept above 36.5 °C despite the changing of isoflurane maintenance concentration. (B) The overall respiratory rates decreased at higher (2.0%) isoflurane concentrations.

The effects of changing isoflurane concentrations on physiological parameters (e.g., body temperatures in °C and respiratory rates in breaths/min) are shown in Fig. 7A and 7B, respectively. Although normothermia was maintained (between 36.5 and 38 °C) throughout the experiment, the respiratory rates for all mice decreased following the change of isoflurane concentration.

## Method validation

Our minimally-invasive method for recording EEG signals from mice under isoflurane anesthesia produced data able to be analyzed for concentration-dependent changes in EEG spectral features and EEG discontinuities. This technique does not involve animal surgery and can measure global electrical activity from the animal's cerebral cortex useful to researchers interested in transitions among conscious states. Common signal conditioning methods (e.g., digital signal processing) typically applied to raw EEG recordings reliably reduced the contribution of physiological artifacts and voltage drifts in our system.

It is not unexpected that increasing the delivery of anesthetic agents will cause neurophysiologic changes representing decreased communication among cortical regions [[24], [25], [26]]. Nevertheless, as a verification of our method's capacity to track EEG changes in response to anesthesia, we observed a decrease in the overall EEG amplitude upon transitioning from the 1.5% to 2.0% isoflurane concentration. This was also associated with a slowing of the oscillatory activity of the EEG, as per the results shown in Figs. 4A and 5A. Furthermore, anesthesia induced inactivation of cortical areas can lead to BS [27], which, using the EEG collected from our anesthesia protocol and designated method, we demonstrate as an increase in the discontinuities observed in EEG. As demonstrated in Fig. 6, this is seen from the increase in BSR after an increase in isoflurane concentration. BS can be clearly observed from EEG as a global cortical phenomenon. Steriade et al. and Marcuse et al. reported from experimental investigations that during the suppression period of anesthesia-induced BS, approximately 95% of cortical neurons become hyperpolarized and then electrically silent during periods of EEG suppression due to inhibition of cortical activity via GABA-ergic mechanisms [[28], [29], [30]]. This results from increased inhibition at cortical synapses, which collectively propagates functional disconnection of the cortex from its thalamic input [30]. On the other hand, during the bursts of BS, breakthrough EEG activity occurs primarily due to intact glutaminergic transmission [29]. Nevertheless, due to an increase in anesthesia induced cortical inhibition, we observed BS with more extensive periods of isoelectricity (increased BSR) during exposure to the 2.0% isoflurane concentration, so we are confident that our system accurately detects this hyperpolarization of cortical neurons.

We found that during 2.0% isoflurane maintenance, the EEG captured by our method had significantly lower power over the entire frequency range compared to the EEG during 1.5% isoflurane maintenance, while the relative PSD between the two groups remained approximately the same. This suggested that despite profound suppression of synaptic activity when oscillations were present, the relative contribution of each relevant frequency bands to the EEG waveform was comparable. In other words, the overall structure of the EEG remained comparable, despite anesthesia-induced EEG suppression. This collectively indicated that while the recorded signal consisted of various artifacts (i.e., line noise and physiological artifacts), the expected EEG changes as a response to the effects of anesthetic hypnosis were prevalent.

The paramount advantages of our developed system include: 1) lower cost, 2) rapid set-up time and facility to repeat recordings (i.e., simple insertion or re-insertion of needle electrode sensors), 3) avoidance of surgery for implantation of electrodes (i.e., reduction of inflammation or infection and more user friendly for anesthesiology researchers with lack of neuroscientific expertise), 4) modifiability of the system (i.e., clipping of needle electrodes for smaller animals or adjusting sampling frequency resolution), 5) simplification of sensors (i.e., non-invasiveness and non-implantable), and 6) allows for natural movement of animals (i.e., animal movement during anesthesia emergence). However, the disadvantages of our system are 1) instability in freely moving animals (i.e., the needle electrode may easily get detached) and 2) an inability to distinguish specific cortical regions with anatomical precision in the recordings. However, we were less interested in the anatomical specificity for neurophysiologic recordings as no one particular lobe or cortical structure governs quiescent states. If anatomical specificity is not necessary, our method could be both advantageous and reliable to collect EEG information representative of global conscious states during anesthesia, sleep, or other neuroscientific research. Therefore, this method is ideally suited for pharmacologic and neurophysiologic investigations of analgo-sedative agents.

### Method limitations

Unfortunately, like most EEG recording systems, noise could not be completely eliminated with our methodology. The source of substantial DC voltage drifts is likely due to our choice to use needle electrodes, which have higher electrode impedances compared to surface electrodes. Furthermore, the use of needle electrodes also meant that our system was sensitive to surrounding electromagnetic noise. For example, interference from personal communication devices can have heterogenous effects on the signal-to-noise ratio for each EEG recording. Subsequently, the signal quality in our system influenced our method of BSR extraction by making automated analysis impossible. Other recording systems may produce EEG recordings with reduced sensitivity to induced noise by using built-in preamplifiers (e.g., active electrodes), improved shielding of the cables [31], and a small Faraday cage. Of course, these modifications would require a higher cost. Additionally, despite our employment of digital signal processing tactics, some of our EEG recordings remained contaminated by respiratory and myogenic artifacts. Even after filtering, these are notoriously difficult to completely eliminate in any EEG recording setup. We did not examine every possible solution (e.g., wavelet analysis) to eliminate these artifacts. In terms of reducing the physiological artifacts (i.e., cardiac or respiratory events), it is a possibility to improve this method by subtracting recorded EMG and respiratory traces from the collected EEG trace.

Because we were unable to measure brain or plasma concentrations of our delivered anesthetic agents, it is possible that our two experimental conditions reflect transitions among concentrations rather than steady state brain concentration. However, because the change in respiratory rate was nearly instantaneous, we feel that any inaccuracy in isoflurane dose has minimal contribution to our results. In addition, at these gas flows and respiratory rates, it could be assumed that isoflurane equilibration occurs in only a few minutes [32]. Although determining the concentration-response of BS was not a focus of this manuscript, it is possible that other physiologic aberrations related to gas exchange (i.e., acidosis and hypoxia) may have contributed to burst suppression in our experiments. We did not specifically measure acid-base status or dissolved oxygen, however based on the widespread use of these isoflurane concentrations in published literature, we feel it is safe to assume the animals were in the normal physiologic range [[33], [34], [35]].

## Conclusion

Here, we described a convenient neuroscience of anesthesia focused methodology for obtaining wireless EEG recordings in anesthetized mice with less invasiveness and less cost. The study of cortical neurophysiological phenomena leading to specific electrical patterns under anesthesia can be efficiently investigated with this system. Using this method, we verified that the delivery of higher concentrations of isoflurane anesthesia are associated with a decrease in EEG amplitude and a decrease in cortical activity as evidenced by a decrease in EEG power and an increase in BSR (increase in isoelectric suppressions and EEG discontinuities due to BS). Furthermore, we verified that the relative distribution of frequency spectra remains essentially unchanged with an increase in isoflurane concentration. Although these results are expected given a well-established body of work in human EEG recorded while receiving anesthesia [23,27,28], our results can be considered confirmatory in a simplified, pre-clinical model, using a more user-oriented methodology.

## CRediT authorship contribution statement

**Mohammad T. Mansouri:** Methodology, Investigation, Resources, Writing – original draft, Writing – review & editing, Project administration. **Meah T. Ahmed:** Software, Conceptualization, Formal analysis, Data curation, Writing – original draft, Writing – review & editing, Visualization. **Tuan Z. Cassim:** Investigation, Data curation, Writing – review & editing. **Matthias Kreuzer:** Conceptualization, Software, Formal analysis, Writing – review & editing, Visualization. **Morgan C. Graves:** Investigation, Writing – review & editing. **Thomas Fenzl:** Conceptualization, Validation, Writing – review & editing. **Paul S. García:** Conceptualization, Methodology, Validation, Resources, Writing – review & editing, Supervision, Funding acquisition.

## Declaration of Competing Interest

The authors declare that they have no known competing financial interests or personal relationships that could have appeared to influence the work reported in this paper.

## Data Availability

Data will be made available on request.
